# Dysregulation of GABAergic Signaling in Neurodevelomental Disorders: Targeting Cation-Chloride Co-transporters to Re-establish a Proper E/I Balance

**DOI:** 10.3389/fncel.2021.813441

**Published:** 2022-01-05

**Authors:** Enrico Cherubini, Graziella Di Cristo, Massimo Avoli

**Affiliations:** ^1^European Brain Research Institute (EBRI)-Rita Levi-Montalcini, Roma, Italy; ^2^Neurosciences Department, Université de Montréal and CHU Sainte-Justine Research Center, Montreal, QC, Canada; ^3^Montreal Neurological Institute-Hospital and Departments of Neurology and Neurosurgery and of Physiology, McGill University, Montreal, QC, Canada

**Keywords:** GABAergic signaling, cation-chloride co-transporters, neurodevelopmental disorders, neuronal oscillations, E/I balance, autism spectrum disorders, schizophrenia, epilepsy

## Abstract

The construction of the brain relies on a series of well-defined genetically and experience- or activity -dependent mechanisms which allow to adapt to the external environment. Disruption of these processes leads to neurological and psychiatric disorders, which in many cases are manifest already early in postnatal life. GABA, the main inhibitory neurotransmitter in the adult brain is one of the major players in the early assembly and formation of neuronal circuits. In the prenatal and immediate postnatal period GABA, acting on GABA_A_ receptors, depolarizes and excites targeted cells *via* an outwardly directed flux of chloride. In this way it activates NMDA receptors and voltage-dependent calcium channels contributing, through intracellular calcium rise, to shape neuronal activity and to establish, through the formation of new synapses and elimination of others, adult neuronal circuits. The direction of GABA_A_-mediated neurotransmission (depolarizing or hyperpolarizing) depends on the intracellular levels of chloride [Cl^−^]_i_, which in turn are maintained by the activity of the cation-chloride importer and exporter KCC2 and NKCC1, respectively. Thus, the premature hyperpolarizing action of GABA or its persistent depolarizing effect beyond the postnatal period, leads to behavioral deficits associated with morphological alterations and an excitatory (E)/inhibitory (I) imbalance in selective brain areas. The aim of this review is to summarize recent data concerning the functional role of GABAergic transmission in building up and refining neuronal circuits early in development and its dysfunction in neurodevelopmental disorders such as Autism Spectrum Disorders (ASDs), schizophrenia and epilepsy. In particular, we focus on novel information concerning the mechanisms by which alterations in cation-chloride co-transporters (CCC) generate behavioral and cognitive impairment in these diseases. We discuss also the possibility to re-establish a proper GABA_A_-mediated neurotransmission and excitatory (E)/inhibitory (I) balance within selective brain areas acting on CCC.

## Introduction

**Figure 1:**
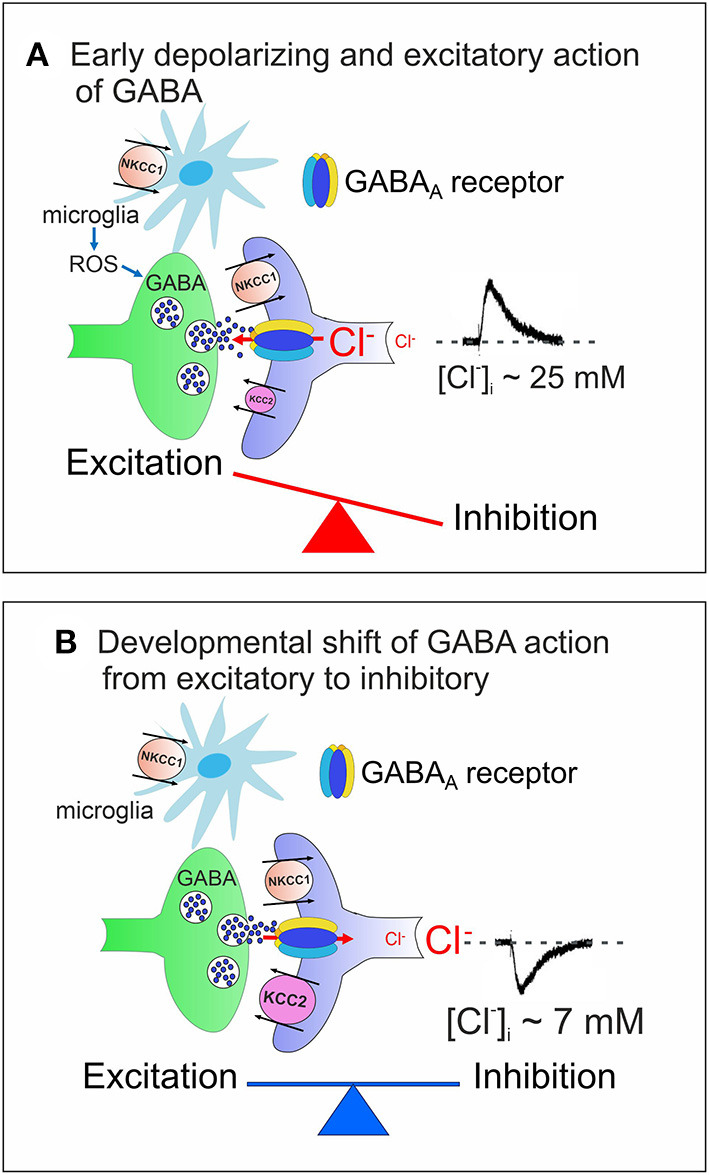
Cation chloride co-transporters contribute to maintain a proper E/I balance in neuronal circuits. **(A)** Early in postnatal life, in rodent's hippocampus, GABA, released from GABAergic interneurons, exerts *via* GABA_A_ receptors a depolarizing, excitatory action in targeted cells by an outwardly directed flux of Cl^−^. High [Cl^−^]_I_ results from the differential temporal expression of Cl^−^ importer and exporter NKCC1 and KCC2, respectively, and accumulation of Cl^−^ inside *via* NKCC1. Early in postnatal life, GABAergic transmission is controlled also by microglia *via* ROS. The early depolarizing and excitatory action of GABA, which leads to E/I unbalance, is instrumental in stimulating synaptogenesis and in shaping early neuronal circuits. **(B)** In juvenile and adult animals, the upregulated expression of KCC2 contributes to maintain a low [Cl^−^]_I_, responsible for the hyperpolarizing action of GABA and for preserving a proper E/I balance in neuronal circuits. In **(A,B)** both pre- (GABAergic interneurons, green) and post-synaptic (targeted cells, violet) elements are represented. Red arrows indicate the direction of Cl^−^ flux through GABA_A_ receptor channels.

In the adult mammalian central nervous system (CNS), γ-aminobutyric acid (GABA) inhibits neuronal firing by activating two different classes of receptors: GABA_A_ and GABA_B_. While GABA_A_ receptors are integral ion channels, GABA_B_ receptors are coupled to ion channels *via* guanine nucleotide-binding proteins and second messengers. The opening of GABA_A_ receptor channels by GABA leads to an inwardly directed flux of Cl^−^ that, by hyperpolarizing the membrane, inhibits neuronal firing. Early in postnatal life, instead, GABA, *via* GABA_A_ receptors, depolarizes and excites targeted cells by an outwardly directed flux of Cl^−^ (Ben-Ari et al., 1989). This phenomenon is due to the high levels of intracellular Cl^−^ ([Cl^−^]_i_) that result from the differential temporal expression of the cation-chloride co-transporters NKCC1 and KCC2, which are involved in Cl^−^ uptake and extrusion, respectively. The low expression of the KCC2 extruder at birth leads to Cl^−^ accumulation inside the neuron *via* NKCC1. The developmentally up-regulated expression of KCC2, which in rodents occurs toward the end of the first postnatal week, results in the extrusion of Cl^−^, causing the shift of GABA from depolarizing to hyperpolarizing direction (Rivera et al., 1999) (Figure 1).

At the network level, the interplay between the depolarizing action of GABA and glutamate generates a primordial form of synchrony which varies in its specific patterns among different brain regions (Buzsáki and Draguhn, 2004; Griguoli and Cherubini, 2017). In the hippocampus, the so-called Giant Depolarizing Potentials (GDPs), are crucial for synaptic wiring and refinement of local neuronal circuits (Ben-Ari et al., 2012). Principal neurons are driven by GABAergic interneurons (Mohajerani and Cherubini, 2005), which act as functional hubs to synchronize large neuronal ensembles (Bonifazi et al., 2009). Early Ca^2+^ signals associated with GDPs act as coincident detectors for enhancing synaptic efficacy at emerging GABAergic (Kasyanov et al., 2004) and glutamatergic synapses (Mohajerani et al., 2007). GDPs are indeed instrumental in converting silent synapses into active ones (Kasyanov et al., 2004), a key mechanism for persistently increasing synaptic efficacy (Voronin and Cherubini, 2004). Immediately after birth, at least in the rodent CA3 hippocampal region, GDPs are associated to intrinsic bursts, driven by a persistent Na^+^ current (Sipilä et al., 2006) and are facilitated by the low expression of Kv7.2/Kv7.3 channels, responsible for the non-inactivating, low-threshold M current (I_M_) (Safiulina et al., 2008). GDPs disappear toward the end of the first postnatal week, when the polarity of GABA shifts from depolarizing to hyperpolarizing.

It is therefore not surprising that the depolarizing action of GABA at early stages of postnatal development coincides with the period of maximal synaptogenesis (Huttenlocher, 1979; De Felipe et al., 1997; Virtanen et al., 2018). Interestingly, GABAergic signals operate before glutamatergic ones, which appear later in concomitance with the development of dendritic arborization (Tyzio et al., 1999; Khazipov et al., 2001; Ben-Ari et al., 2007). The late switch of GABA polarity at the axon initial segment of principal cells favors more organized forms of network oscillations such as those occurring in the gamma range (20–80 Hz), as demonstrated in the somatosensory (Khirug et al., 2008) and in the prefrontal cortex (Rinetti-Vargas et al., 2017). The early depolarizing action of GABA and its developmental shift, mainly documented in *in vitro* studies, have been challenged because of the lack of direct *in vivo* demonstrations (Ben-Ari et al., 2012). Using a combined electrophysiological and imaging techniques from anesthetized neonatal mice, it has been shown that, in spite of its depolarizing action, GABA inhibits cortical activity *via* a shunting inhibitory action (Kirmse et al., 2015).

Similarly, an inhibitory effect of GABA on spontaneous glutamatergic events has been reported in the hippocampus of anesthetized animals during the first postnatal week, following photo-stimulation of GABAergic interneurons expressing channelrhodopsin (Valeeva et al., 2016).

Evidence for an early depolarizing and excitatory action of GABA *in vivo* has been provided by Oh et al. (2016) who have demonstrated that, in the developing mouse cortex, synapses formation requires GABA-mediated activation of T-type voltage dependent Ca^2+^ channels. The early depolarizing and excitatory action of GABA in *in vivo* conditions has been further confirmed by Sulis Sato et al. (2017) and Murata and Colonnese (2020). Using a particular probe formed by the fusion of a Cl^−^ and pH-sensitive GFP mutant with an ion-insensitive red fluorescent protein, which allows the combined measurement of [Cl^−^]_I_ and pH, Sulis Sato et al. (2017) have demonstrated, by means of two-photon *in vivo* imaging from individual pyramidal cells in the mouse cortex, the developmental shift of GABAergic signaling; this effect could be mimicked by the selective NKCC1 antagonist bumetanide. In addition, using chemogenetic and optogenetic approaches, Murata and Colonnese (2020) have proved that, at postnatal day 3 in non-anesthetized mice, GABA released from GABAergic interneurons increases the firing of CA1 principal cells. However, according to these authors, the shift of GABA polarity is region-specific, since at the same age GABAergic interneurons exert an inhibitory action on visual cortex principal cells.

Our review will focus on evidence concerning the functional role of GABAergic signaling in brain maturation and its alterations in neurodevelopmental disorders, highlighting the contribution of CCC to these effects. CCC are intrinsic membrane proteins that transport Cl^−^ ions, together with Na^+^ and/or K^+^ ions, in an electroneutral manner due to the stoichiometric coupling and directionality of translocated ions. Therefore, members of this family are prime regulators of [Cl^−^]_i_. After the initial discovery of the depolarizing action of GABA (Ben-Ari et al., 1989; Cherubini et al., 1991), a fundamental step forward to understanding why in immature neurons the equilibrium potential for chloride (${\text{E}}_{\text{C}{\text{l}}^{-}}$) is positive relative to the resting membrane potential (V_m_) has been made by Rivera et al. (1999) who demonstrated that the developmental regulated expression of the cation-chloride exporter KCC2 leads to Cl^−^ accumulation inside young neurons with consequent shift of GABA action from hyperpolarizing to depolarizing. It is worth mentioning that GABA_A_ receptor channels are permeable not only to Cl^−^ but also to ${\text{HCO}}_{3}^{-}$ and therefore, the equilibrium potential for Cl^−^ (${\text{E}}_{\text{C}{\text{l}}^{-}}$) does not correspond precisely to the equilibrium potential of GABA (E_GABA_), which usually shifts toward more positive values, being E_HCO3−_ less negative than ${\text{E}}_{\text{C}{\text{l}}^{-}}$ (Kaila, 1994). Taking into account a HCO3^−^/Cl^−^ permeability ratio of 0.2–0.4, the quantitative influence of ${\text{HCO}}_{3}^{-}$ on E_GABA_ can be estimated using the Goldman-Hodgkin-Katz equation, which shows that the [${\text{HCO}}_{3}^{-}$]_i_ (~15 mM at a pH of 7.1–7.2) influences E_GABA_ in the same way as 3–5 mM of [Cl^−^]i. Thus, as compared to adult neurons, in the immature CNS, in which [Cl^−^]_i_ is relatively high, the depolarizing influence of ${\text{HCO}}_{3}^{-}$ on E_GABA_ is negligible. In addition to controlling E_GABA_, the direction of GABA action and network excitability, CCCs regulate many physiological processes including cell volume, water transport and intracellular pH (Delpire and Gagnon, 2018).

## Distribution of NKCC1 and KCC2

Encoded by the *SLC12* gene's family, CCCs are glycoproteins that are widely distributed in all organ systems, including the brain (Kaila et al., 2014). Among CCCs, the main chloride extruder KCC2 has been found almost exclusively in the CNS. Consistent with a developmental gradient, at birth KCC2 is already present in the spinal cord and in the brainstem in rodents, while it starts to be upregulated later in most rostral regions of the brain (Kaila et al., 2014; Virtanen et al., 2020). In the human cortex, upregulation of KCC2 starts prenatally from the 25th postconceptional week and peaks at birth (Sedmak et al., 2016). In preterm infants, the relatively low expression of KCC2 is associated with a discontinuous type of EEG—organized in intermittent bursts of activity, separated by silent periods reminiscent of GDPs—that disappears at birth (Khazipov and Luhmann, 2006). It is worth noting that KCC2 has been also detected in pancreatic β-cells where it plays a crucial role in modulating insulin secretion (Kursan et al., 2017).

In the hippocampus, KCC2 is involved in regulating GDPs, which are driven by the synergistic depolarizing action of glutamate and GABA (Bolea et al., 1999; Ben-Ari et al., 2012) and by the intrinsic pacemaker properties of CA3 pyramidal neurons (Strata et al., 1997; Safiulina et al., 2008; Griguoli and Cherubini, 2017). Interestingly, during the first week of postnatal life, using whole-cell Cl^−^ loading experiments, Spoljaric et al. (2019) have reported that the selective KCC2 antagonist VU0463271 can increase the firing rate of CA3 principal cells as well as their synchrony during the rising phase of GDPs, suggesting that these neurons are able to actively extrude Cl^−^ in a KCC2-dependent way, particularly when GABA is applied at the dendritic level. In addition to its canonical function of transporting Cl^−^ outside the neuron, KCC2 plays a key role in controlling actin cytoskeleton's dynamics and spinogenesis at early developmental stages (Blaesse et al., 2009; Virtanen et al., 2020). Of note, during the first postnatal week, KCC2 promotes spinogenesis independent of KCC2 Cl^−^ transport function in the somatosensory cortex (Li et al., 2007; Fiumelli et al., 2013), but, at the same age, it constrains spine density in hippocampal CA1 neurons, an effect that is instead dependent on the transporter function (Awad et al., 2018). This difference might be explained by distinct membrane localizations of KCC2 in different neuron types or/and brain region during early postnatal development. Nevertheless, it is safe to state that KCC2 plays multiple roles in brain development, contributing to shape neuronal circuits shortly after birth and network plasticity at later stages of postnatal development (Virtanen et al., 2021).

Unlike KCC2, the main Cl^−^ importer NKCC1 is expressed in the brain already at birth, where it plays a key role in maintaining high [Cl^−^]_i_ in immature neurons. NKCC1 is widely distributed not only in CNS and in the peripheral nervous system (PNS) but also in a variety of different tissues including the inner ear, in skeletal and smooth muscles, exocrine glands, epithelial cells and kidneys, where it contributes to regulate major physiological functions (Delpire and Gagnon, 2018; Virtanen et al., 2020). While in the CNS, the majority of neurons express low levels of NKCC1, in the PNS sensory neurons such as dorsal root or trigeminal ganglion cells, exhibit high amounts of the protein. When GABA from local interneurons is released at the terminals of sensory afferent fibers, it causes a membrane depolarization that leads to a reduction of glutamatergic transmission and to an inhibitory response. This effect is reduced in NKCC1 knock-out mice, indicating that the depolarizing value of E_GABA_ is maintained *via* NKCC1. Such mechanism may have a strong implication for nociception, as demonstrated by deficits in thermal nociceptive threshold in NKCC1 knock-out mice (Sung et al., 2000). Primary sensory afferents (i.e., dorsal root ganglion fibers) contain GABA receptors, whose activation by GABA, released from interneurons localized in lamina I/II of the dorsal horn, causes a depolarization (primary afferent depolarization or PAD). The depolarization, maintained by the expression of NKCC1 and the lack of KCC2 (Alvarez-Leefmans et al., 2001) leads to suppression of nociceptive signals mainly *via* inactivation of voltage-gated Na^+^ channels and consequent reduction of transmitter release (Price et al., 2009) An inflammatory insult, after peripheral nerve injury, may cause an upregulation of NKCC1 activity in nociceptive afferent fibers leading to an increased [Cl^−^]_i_ and an excessive GABA_A_-mediated depolarization that would facilitate cross-excitation between low and high threshold nociceptive afferent fibers and nociception (Price et al., 2005).

In the CNS, the selective deletion from hippocampal CA1 pyramidal cells of the *SLC12a2 gene*, leads to an attenuation of the depolarizing action of GABA, due to a reduction of [Cl^−^]_i_ and to a severe impairment of GDPs activity, in a region-specific way (Graf et al., 2021). However, such deletion only slightly affects the *in vivo* network dynamics or hippocampal-dependent behavioral tasks, suggesting that most of the effects observed in NKCC1 knock-out mice or after pharmacological blockade of the transporter, may be attributed to the loss of the protein from non-neuronal cells or from cells localized outside the brain (Graf et al., 2021). In the brain, NKCC1 has been found to be expressed in several non-neuronal cell types such as choroid plexus epithelial cells, astrocytes, oligodendrocytes and microglia (Tóth et al., 2021). In particular NKCC1 is highly expressed in microglia (DePaula-Silva et al., 2019) (Figure 1) where it plays a fundamental role in neuro-inflammation. Thus, the selective deletion of NKCC1 on microglia affects their cell volume and baseline morphology and boosts cytokines production in response to inflammatory stimuli (Tóth et al., 2021). Interestingly, it has been recently reported that long-term potentiation triggers in potentiated synapses withdrawal of perisynaptic astrocytic processes, which involves the NKCC1 transporter and the actin-controlling protein cofilin. This favors glutamate spillover and NMDA-mediated inter synaptic cross-talk, crucial for LTP and memory formation (Henneberger et al., 2020).

## Transcriptional and Post-translational Regulation of NKCC1 and KCC2

Among transcriptional regulators of CCCs, a key factor is represented by Brain Derived Neurotrophic Factor (BDNF) and its tropomyosin kinase B (TrkB) receptor. Employing transgenic embryos that overexpress BDNF under the control of the nestin promoter, Aguado et al. (2003) demonstrated that, in embryonic hippocampal slices, BDNF powerfully controls the developmental switch of GABAergic transmission. At the network level, by upregulating KCC2 expression, BDNF reduced [Cl^−^]_i_ and GABA_A_-activated Ca^2+^ transients. Furthermore, in immature cultured hippocampal neurons, BDNF enhanced KCC2 mRNA and protein expression levels *via* ERK1/2-dependent upregulation of Egr4 transcription factor (Ludwig et al., 2011a). BDNF can further increase KCC2 activation, by promoting the localization at the membrane of already synthetized KCC2 in the developing brain (Khirug et al., 2010; Puskarjov et al., 2015; Awad et al., 2018). Egr4 mRNA expression can be also triggered by the trophic factor neurturin which leads to the developmental upregulation of KCC2 in an ERK1/2-dependent way (Ludwig et al., 2011b). Furthermore, TrkB-deficient mice exhibit a reduced number of GABAergic synapses associated with decreased expression levels of KCC2, further indicating that BDNF is determinant for its expression (Carmona et al., 2006). These data are inconsistent with those reported by Puskarjov et al. (2015) on BDNF-deficient mice in which no developmental changes in GABA shift were detected. Although the lack of the developmental upregulation of KCC2 in BDNF null mice may be related to compensatory processes, the reason for this discrepancy is still unclear.

While the impact of BDNF/TrkB signaling on KCC2 expression at early stages of postnatal development has been well-documented, the role of this neurotrophin on NKCC1 expression is still debated. A recent study by Badurek et al. (2020) has however unveiled that the selective deletion of TrkB from immature dentate granule cells (DGC), when these cells integrate the hippocampal circuit, induces a premature shift of GABA from the depolarizing to the hyperpolarizing direction at mossy fibers-CA3 synapses, which at birth are GABAergic (Safiulina et al., 2006a). A dysfunction in BDNF/TrkB signaling leads due to downregulation of NKCC1 expression and low [Cl^−^]_i_, in the absence of any effect on KCC2. In agreement with a previous study on immature neocortical neurons (Cancedda et al., 2007), the premature hyperpolarizing shift of GABA prevents the establishment of proper synaptic connectivity in targeted neurons, an effect that persists in adulthood (Badurek et al., 2020). However, how in immature DGCs, BDNF/TrkB signaling regulates the expression of the Cl^−^ importer NKCC1 remains to be elucidated. Another trophic factor that controls KCC2 expression and the developmental GABA switch is the insulin growth factor 1 (IGF-1), which presumably requires protein tyrosine kinase c-Src (Kelsch et al., 2001). It is worth noting that, independently on its action on CCCs, the BDNF/TrkB signaling pathway is instrumental in tuning hippocampal wiring at emerging GABAergic (Sivakumaran et al., 2009) and glutamatergic (Mohajerani et al., 2007) synapses, during spike time dependent plasticity, an Hebbian form of learning.

Interestingly, neuroligin 2 (NLG2) a cell adhesion molecule involved in regulating GABAergic synaptogenesis has recently emerged as a key modulator of the developmental GABAergic switch. It was unexpectedly discovered that, knocking down NLG2, leads to a reduced expression of KCC2, which is in turn associated with a delayed switch of GABA from the depolarizing to the hyperpolarizing direction. The down-regulation of KCC2 was accompanied by a reduced number of dendritic spines and glutamatergic synaptic events, suggesting that, in neural networks, NLG2 may serve as a master regulator of the delicate balance between glutamatergic and GABAergic functions (Sun et al., 2013).

Among post-translational mechanisms controlling the activity and stabilization of CCC, protein phosphorylation represents the main functional substrate. KCC2 and NKCC1 are regulated in a reciprocal fashion by threonine-dependent phosphorylation/de-phosphorylation residues, targeted by WNK (with-No-Lysine) kinases that are responsible for increasing or decreasing [Cl^−^]_i_ levels, respectively (Ben-Ari et al., 2012; Kahle et al., 2013; Kaila et al., 2014). The reciprocal activation is probably determined by similar four amino acid phosphorylation motifs that are present on C and N terminus domains of KCC2 and NKCC1, respectively (Rinehart et al., 2009). Interestingly, early in postnatal life, oxytocin, a hypothalamic hormone known to promote parturition and lactation and to be involved in social behavior, regulates GABA switch by upregulating the activity of KCC2, through the promotion of its phosphorylation at Ser940 and its insertion at the plasma membrane without impairing NKCC1 (Leonzino et al., 2016).

## Involvement of NKCC1 and KCC2 in Maintaining a Proper Ratio Between Excitation and Inhibition Within Neuronal Circuits

By regulating the direction of GABA action and therefore the efficacy of inhibition, NKCC1 and KCC2 contribute to set a proper E/I balance within selective neuronal circuits (Figure 1B). A proper ratio between excitation (E) and inhibition (I), the so-called E/I balance is thought to be critical for controlling spike rate and information processing. It requires precise connections through dynamic processes involving neurotransmitter receptors, transporters, scaffolding proteins, and the cytoskeleton. Using an open source software to map the distribution and morphology of excitatory and inhibitory synapses along the dendritic tree of layer 2/3 mouse cortical pyramidal neurons with computational modeling, Iascone et al. (2020) unveiled that E/I synapses are highly regulated by molecular mechanisms operating locally to generate a relative invariant E/I ratio across dendritic segments.

Failure to maintain a proper E/I balance within key neuronal circuits is thought to account for behavioral deficits observed in several neurological diseases (Yizhar et al., 2011; Lee et al., 2017; Ghatak et al., 2021). A reduced inhibition or an excessive excitation may cause an increased signal to noise ratio with consequent neuronal hyper-excitability and seizures. Conversely, an enhanced inhibition may lead to a reduced signal to noise ratio and to a lower level of activity (Sohal and Rubenstein, 2019). Both conditions would affect information processing. Changes occurring at synaptic and circuit levels would influence the interplay between GABAergic interneurons and targeted pyramidal cells leading to altered temporal integration and abnormal rhythmogenesis. In cortical circuits, the E/I balance plays a critical role in regulating the responses of neuronal circuits to sensory stimuli. Thus, in juvenile mice carrying the human R451C mutation of the gene encoding for neuroligin 3 (an adhesion molecule essential for synaptic stabilization) found in some families with children affected by Autism Spectrum Disorders (ASDs), the impairment of GABA release from parvalbumin (PV)+ basket cells was found to severely alter the E/I balance in layer IV neuronal microcircuit of the somatosensory cortex (Cellot and Cherubini, 2014a). This represents a critical issue, since PV+ cells, which are innervated by the same thalamic afferents to excitatory layer IV spiny neurons, play a crucial role in sensory information, acting as an inhibitory gate for incoming thalamic inputs *via* feed-forward disynaptic inhibition (Cellot and Cherubini, 2014a). Changes in the inhibitory gate may alter sensory processing in ASD patients leading to misleading sensory representations with difficulties to combine pieces of information into a unified perceptual whole.

Although an E/I imbalance has been implicated in various brain disorders, this concept is rather broad and oversimplified. It should be used indeed with caution, particularly in view of our progress in understanding, thanks also to the development of optogenetics, the functional role of selective neuronal circuits in behavior. Both excitation and inhibition are not unidimensional entities but originate from multiple sources, which are dynamically regulated in space and time (He and Cline, 2019). Differences in local circuits connectivity, can produce various levels of inhibition or disinhibition in different pathways. Distinct classes of cortical GABAergic interneurons may differently contribute to seizures, by suppressing or prolonging them (Khoshkhoo et al., 2017). Therefore, in these cases a therapeutic intervention directed against a particular type of interneuron will be more effective than one aimed at inhibition in general.

CCC dynamically regulate in an activity-dependent way GABA_A_-mediated synaptic strength (Woodin et al., 2003; Fiumelli and Woodin, 2007). Hence, brief synaptic stimulations or pairing pre- and postsynaptic activity induce long-term synaptic plasticity changes at GABAergic synapses, exhibiting a positive shift in (E_GABA_), mediated by a decrease in KCC2's function and increase of [Cl^−^]_i_, with consequent decline of synaptic inhibition (Balena and Woodin, 2008). Activity-dependent changes in E_GABA_ requires Ca^2+^ influx through voltage-gated calcium channels and NMDA receptors (Balena and Woodin, 2008). CCC also very labile, and they can be disrupted in several neuropsychiatric disorders particularly in those originating early in developmental such as ASDs, schizophrenia and epilepsy (Fiumelli and Woodin, 2007). In the following sections, an outline of the involvement of GABAergic signaling in these disorders will be discussed in line with possible therapeutic interventions aimed at targeting CCC to re-establish a proper E/I balance at synaptic level and/or neuronal connectivity at the circuit level.

## Autism Spectrum Disorders

**Figure 2:**
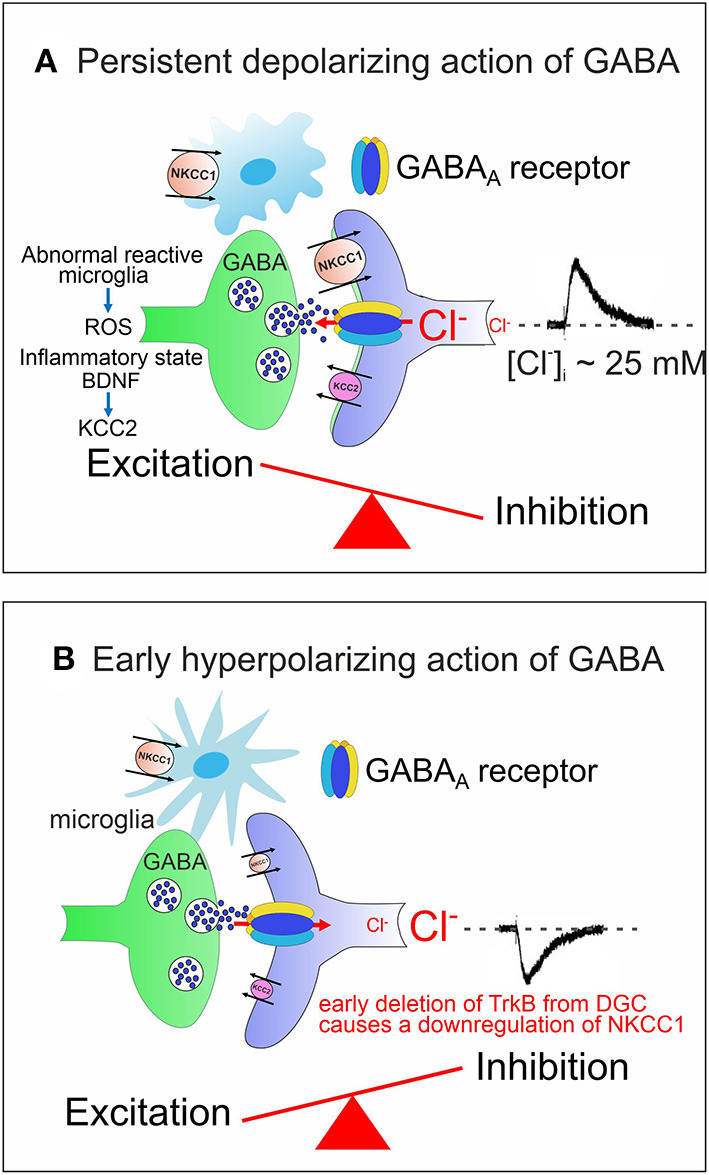
Alterations in developmental GABA shift lead to neuropsychiatric disorders. **(A)** The persistent depolarizing action of GABA beyond the critical period, impairs network excitability and the E/I balance. Abnormal reactive microglia, produces high levels of ROS, often associated with an inflammatory state caused by a dysfunction of the immune system. BDNF, released from microglia contributes to downregulate KCC2 with consequent enhancement of network excitability. **(B)** The early hyperpolarizing action of GABA at birth, caused by the reduced expression of NKCC1, following deletion of TrkB from immature DGCs, at the time when these cells integrate the classical tri-synaptic pathway, severely alters the morphology and circuitry downstream of DGCs. In **(A,B)** both pre (GABAergic interneurons, green) and post synaptic (targeted cells, violet) elements are represented. Red arrows indicate the direction of Cl^−^ flux through GABA_A_ receptor channels.

ASDs comprise a heterogeneous group of neurodevelopmental disorders characterized by impaired social interactions, deficits in verbal and non-verbal communication, restricted interests and stereotyped behaviors with high incidence (~1/70 children) and a significant economic and social burden for families and society. Impaired chloride homeostasis with consequent changes in the direction of GABA shift during time-sensitive windows, may account for behavioral alterations found in some animal models of ASDs, reminiscent of those observed in autistic patients (Pizzarelli and Cherubini, 2011; Cellot and Cherubini, 2014b). GABA-mediated enhancement in network excitability may account for the high co-morbidity of ASDs with epilepsy (Frye et al., 2013; Bozzi et al., 2018; Sierra-Arregui et al., 2020) and for the paradoxical action exerted by benzodiazepines in some ASD patients (Marrosu et al., 1987). The E/I imbalance in selective brain areas may result either from the persistent depolarizing and excitatory action of GABA beyond the critical period (Tyzio et al., 2014; Corradini et al., 2018; Fernandez et al., 2019) (Figure 2A), or from the early hyperpolarizing action of this neurotransmitter, following downregulation of the chloride importer NKCC1 (Figure 2B).

As already mentioned, the early depolarizing and excitatory action of GABA is essential for shaping neuronal networks, as demonstrated by the morphological impairment of cortical pyramidal neurons (Cancedda et al., 2007) and the premature termination of interneuron migration (Bortone and Polleux, 2009) following precocious KCC2 expression by KCC2 electroporation. A reduced expression of NKCC1 at birth has been recently demonstrated in a novel genetic mouse model (*Ntrk2/Trkb*) in which the selective deletion of TrkB from immature dentate granule cells (DGCs) leads to a disruption of downstream circuits, associated with a severe impairment of synaptic plasticity and cognitive processes (Badurek et al., 2020). BDNF, *via* TrkB signaling pathway, is known to play a crucial role in the maturation of inhibition as demonstrated in cortical and hippocampal neurons (Huang et al., 1999; Yamada et al., 2002). Interestingly, as shown in Figures 1A, 2A, at early stages of brain development, GABAergic signaling is controlled by Reactive Oxygen Species (ROS, Safiulina et al., 2006b), which in ASDs are produced at high levels by abnormal reactive microglia, often associated with a dysfunction of the immune system sustained by a strong inflammatory state (Pangrazzi et al., 2020). The exact mechanisms by which microglia alters the strength of inhibition is still unclear. One possibility is that microglia interacts with CCC *via* the BDNF-TrkB signaling pathway. Thus, BDNF, released from microglia, would cause disinhibition *via* downregulation of KCC2 (Rivera et al., 2002; Coull et al., 2005). This effect may involve KCC2 de-phosphorylation with consequent reduction of surface protein expression and increased protein turnover (Wake et al., 2007). In addition, neuroinflammation associated ROS and inflammatory cytokines would activate NKCC1, thereby enhancing neuronal excitation (Alahmari et al., 2015) (Figure 2A). Therefore, it is reasonable to hypothesize that targeting CCC may allow, at least in some cases, to improve cognitive deficits in ASDs by re-establishing a correct GABAergic signaling in neuronal circuits.

One simple approach to restore physiological levels of [Cl^−^]_i_ is to reduce NKCC1 activity with the selective high affinity antagonist bumetanide. Therefore, this drug has been extensively tested as a potential treatment for a variety of neuropsychiatric disorders (Ben-Ari, 2017). In both syndromic (*Fragile X*) and non-syndromic (valproic acid and maternal immune activation) animal models of ASDs, which are known to be associated with a dysfunction of GABAergic signaling (Tyzio et al., 2014; Corradini et al., 2018; Fernandez et al., 2019), bumetanide, *via* maternal administration, is able to reduce chloride accumulation and to rescue behavioral deficits by re-establishing an appropriate E/I balance in the brain of offspring (Tyzio et al., 2014). Similarly, bumetanide (0.5–2 mg twice daily for 3 months) has been demonstrated to ameliorate cognitive functions in autistic children (Ben-Ari, 2017). Following a pilot study by Lemonnier and Ben-Ari (2010), this drug has been tested by the same group in two placebo-controlled randomized studies from 60 and 88 children, respectively (Lemonnier et al., 2012, 2017). These and other studies on autistic children from different countries (Bruining et al., 2015; Hajri et al., 2019; Zhang et al., 2020; Fernell et al., 2021), have demonstrated beneficial effects of bumetanide on cognitive functions, as assessed by Child Autistic Rating Scale (CARS), Clinical Global Impressions Improvement Scale (CGI-I), Social Responsiveness Scale (SRS), and Aberrant Behavior Checklist (ABC), with only few minor side effects (such as mild hypokalaemia; loss of appetite, diuresis, dehydration, asthenia). In one clinical trial, bumetanide was associated with the Applied Behavior Analysis (ABA) training. In this case, more positive results were obtained in children treated with bumetanide and ABA respect to those treated with the ABA alone (Du et al., 2015). In adolescents with ASDs, chronic treatment with bumetanide has been shown to significantly improve visual recognition of emotive figures and to reduce amygdala activation in response to eye contacts (Hadjikhani et al., 2015). This would allow increasing the time spontaneously spent looking in the eyes to acquire the necessary information for social processing (Hadjikhani et al., 2018). However, in a recent double blind randomized study from 92 participants (Sprengers et al., 2021), bumetanide did not differ from placebo on sociability effects (assessed by SRS) but, unlike placebo, exerted clear positive effects on repetitive behavior, a core symptom of ASDs (measured with the Repetitive Behavior Scale-Revised). Although, the reason for the discrepant results between these studies is unclear, the possibility that the drug may be effective only in some forms of autism whose symptoms are more related to a GABAergic dysfunction, cannot be excluded. The high heterogeneity among ASD patients may explain why a large phase 3 clinical trial performed in 50 centers from 14 different countries failed to reach significant differences between bumetanide- and placebo-treated children as recently announced by a press release from Servier and Neurochlore (France). Bumetanide has also been reported to attenuate autism's traits but not seizures in patients with Tuberous Sclerosis (Van Andel et al., 2020). Overall, bumetanide has been shown to exert a symptomatic action mitigating, at least in a subpopulation of autistic children, the severity of symptoms, which reappear after interruption of the treatment. A limitation however in using bumetanide for treating Neurodevelopmental Disorders lies on the fact that this drug has poor pharmacokinetic properties and low capability to cross the blood brain barrier (BBB) to reach either neuronal or non-neuronal targets (Puskarjov et al., 2014a; Virtanen et al., 2020; Löscher and Kaila, 2021; Tóth et al., 2021). Moreover, an active efflux of bumetanide from the brain to the blood, which involves several transporters expressed at the BBB, including organic anion ones, would contribute to maintain a very low concentration of the drug in the brain (Römermann et al., 2017). This raises the possibility that the observed beneficial effects of bumetanide are related to some still unknown peripheral-central type of communication.

Another alternative approach to attenuate E/I imbalance in ASDs is to use KCC2 activators to selectively enhance the activity of KCC2, which is a key player in Cl^−^ homeostasis and, unlike NKCC1, it is expressed mainly by neurons (Schulte et al., 2018; Virtanen et al., 2021). Targeting KCC2 specificity may prevent adverse side effects occurring in non-neuronal tissues or in other organs as in the case of NKCC1. Among these, the KCC2 analog CLP257 was able, by lowering [Cl^−^]_i_, to restore chloride transport in neurons with reduced KCC2 activity and to alleviate hypersensitivity in a rat model of neuropathic pain (Gagnon et al., 2013). KCC2 expression is known to be upregulated by phosphatases, insulin growth factor I (IGF-1) and 5-hydroxytryptamine type 2A (5-HT_2A_) receptors (Kelsch et al., 2001; Bos et al., 2013; Baroncelli et al., 2017). More recently, by generating a robust high-throughput drug screening platform that allows for the rapid assessment of KCC2 gene expression in genome-edited human reporter neurons, Tang et al. (2019) identified a group of small molecules that are able to increase KCC2 expression (KCC2 Expression-Enhancing Compounds or KEECs). In an animal model of Rett syndrome (*MeCP2* mutant mouse), exhibiting reduced KCC2 activity, these molecules were able, by enhancing KCC2 expression levels, to restore a proper E/I balance and to ameliorate disease-associated respiratory and locomotion phenotypes. This study did not address whether KEECs can ameliorate other deficits in the mutant mice, In particular social behavioral deficits. Nevertheless, these very promising results pave the way to test whether, at preclinical and clinical levels, these drugs may possibly restore cognitive functions in other neurodevelopmental disorders associated with [Cl^−^]_i_ imbalance.

## Schizophrenia

Schizophrenia is a debilitating psychiatric illness affecting 0.5–1% of the global population, which is characterized by positive (hallucinations and delusions), negative (lack of communication, social interaction, motivation), and cognitive symptoms (Lewis and Lieberman, 2000). Although positive symptoms are the most notable feature of this illness, cognitive disturbances are typically present before the onset of psychosis and are the best predictor of long-term functional outcome (Kahn and Keefe, 2013). The affected domains of cognition include working memory, executive function, learning and long-term memory, visual/auditory perception, and attention (Carter et al., 2008). In particular, working memory impairment is thought to be a core feature of schizophrenia, because it can influence all other observed cognitive deficits (Silver et al., 2003).

Working memory function is associated with oscillatory activity in the gamma frequency range (30–80 Hz) in the prefrontal cortex. The power of gamma oscillations in the prefrontal cortex normally increases in proportion to working memory load (Howard et al., 2003; Jensen et al., 2007), but in individuals with schizophrenia this increase is reduced (Uhlhaas and Singer, 2010). Moreover, these deficits have been detected in both individuals with chronic illness (Cho et al., 2006) and in subjects with the first-episode of psychosis (Minzenberg et al., 2010), suggesting that working memory impairments and disrupted gamma power reflect the disease process of schizophrenia and are not due to chronic illness or the use of antipsychotic medications.

Gamma oscillations refer to the synchronous firing of large ensembles of excitatory glutamatergic pyramidal neurons within and across brain regions, which is paced by GABAergic interneurons (Gonzalez-Burgos and Lewis, 2008). In particular, fast spiking PV+ GABAergic interneurons, connected by gap junctions, shape *via* feed-forward inhibition, the spatial and temporal profile of pyramidal cells firing, to functionally impact the information processing (Hu et al., 2014). The coordinated activity of glutamatergic and GABAergic neurons in triggering gamma activity, is commonly referred as the pyramidal interneuron network gamma (PING) (Gonzalez-Burgos and Lewis, 2008). The role of GABAergic cells in the generation and modulation of gamma oscillation is strongly supported by numerous studies using pharmacology-, and more recently optogenetics-, based manipulations both *in vitro* and *in vivo* (Whittington et al., 1995; Traub et al., 1996; Fuchs et al., 2007; Cardin et al., 2009; Sohal et al., 2009). It is thus reasonable to hypothesize that the altered gamma oscillations dynamics observed in schizophrenic individuals may be caused, at least in part, by functional abnormalities in GABA neurotransmission in the prefrontal cortex.

Another observation supporting a putative role for altered GABAergic transmission in schizophrenia is the frequency of co-morbidity of schizophrenia and epilepsy. In fact, both individual and family history of epileptic disorders appear to be associated with schizophrenia (Qin et al., 2005). A Danish population-based cohort study found that a history of juvenile febrile seizures was associated with a 44% increased risk of schizophrenia, while a history of both febrile seizures plus epilepsy was associated with 204% increased risk of schizophrenia (Vestergaard et al., 2005). Overall, it has been estimated that 2–9% of epileptic patients are also diagnosed with schizophrenia compared with an estimated 1% prevalence in the general population (Clarke et al., 2012; Schizophrenia Working Group of the Psychiatric Genomics Consortium, 2014). This comorbidity may be explained by a common genetic architecture between the two disorders; however, this hypothesis needs to be more extensively explored by systematic genome sequencing of patients affected by both epilepsy and schizophrenia. Another potential cause of comorbidity between epilepsy and schizophrenia might be environmental (or non-genetic). For example, neonatal seizures due to global cerebral hypoxia or intracranial hemorrhage are a strong risk factor for later development of both epilepsy and cognitive impairment (Tekgul et al., 2006). Independently of the underlying causes, and as it is the case of ASD, it has been suggested that alterations in GABAergic transmission may account for the co-morbidity of schizophrenia with epilepsy (Kalkman, 2011).

Overall, numerous *in vivo* imaging and postmortem studies have consistently revealed alterations in GABA levels and components of GABAergic circuits in the prefrontal cortex of schizophrenic subjects compared to control cohorts, in particular regarding PV+ basket and chandelier GABAergic cells (extensively reviewed by Dienel and Lewis, 2019). As described above, one of the factors regulating the function of GABA neurotransmission is its nature, which can be hyperpolarizing, depolarizing, or shunting depending on the flow of Cl^−^ ions through GABA-A receptors channels once these are activated. This has prompted researchers to look at the reversal potential of GABA and/or the expression of NKCC1, KCC2 cation-chloride co-transporters and their associated regulatory pathways in mouse models of schizophrenia and post-mortem human tissues.

In the subchronic phencyclidine (scPCP)-treated mice, a well-studied animal model mimicking the cognitive impairment symptoms associated with schizophrenia (Jentsch and Roth, 1999; Steeds et al., 2015; Kim et al., 2021) found that the reversal potential of GABA (E_GABA_) recorded from pyramidal neurons in the infralimbic cortex (the more ventral portion of the prefrontal cortex) was more positive in scPCP-treated mice as compared to those treated with vehicle. This effect was highly specific since it was not found in the prelimbic cortex, which is also part of the prefrontal cortex and sits just above the infralimbic cortex. Changes in E_GABA_ in scPCP mice led to a depolarizing and excitatory action of the neurotransmitter with consequent increase in firing of infralimbic cortex pyramidal neurons that, following a 20 Hz stimulation, generated twice as many action potentials as those from vehicle-treated mice. Using RNAscope *in situ* hybridization, they further reported that NKCC1 mRNAs were increased in infralimbic, but not in prelimbic neurons, of 5 postnatal weeks-old scPCP mice, while KCC2 mRNAs were not altered. Based on this observation, Kim et al. (2021) hypothesized that limiting NKCC1 function may rescue the electrophysiological and behavioral phenotypes. In keeping with this hypothesis, their data showed that intraperitoneal or focal intracortical injection of the selective NKCC1 blocker bumetanide, before initiating behavioral testing, ameliorated scPCP mice performance on different behavioral tasks assessing declarative memory, working memory, and executive function, more specifically in the novel object recognition, Y-maze spontaneous alternation and operant reversal learning tests. Since bumetanide exhibits low brain penetration (Römermann et al., 2017), it has been frequently questioned whether its effects on behavior in different disease animal models was dependent on its specific inhibitory effects on NKCC1 in the brain or rather due to secondary unspecific effects. In the scPCP mouse model, short hairpin RNAi-mediated downregulation of endogenous NKCC1 mimicked the effects of bumetanide on behaviors, therefore supporting the hypothesis that more depolarized GABA reversal potential in the infralimbic cortex pyramidal neurons plays a role in the cognitive deficits observed in this mouse model.

Another evidence supporting the occurrence of dysregulated Cl^−^ balance in schizophrenia-like animal models comes from the study of the *sandy* mouse, a dysbindin null mutation. Dysbindin is encoded by *DTNBP1*. *DTNBP1* polymorphisms are considered risk factors for schizophrenia onset (Straub et al., 2002; Van Den Bogaert et al., 2003), even if a consensus has not been reached yet (Schizophrenia Working Group of the Psychiatric Genomics Consortium, 2014; Farrell et al., 2015). Recent transcriptome studies showed that, in addition to alterations associated with PV+ GABAergic cells, the dysbindin mutant mice showed reduction of both NKCC1 and KCC2 mRNA levels at the late embryonic stage (Larimore et al., 2017).

In humans, clinical genetic studies support an association between NKCC1 (coded by the gene *SLC12A2*) and schizophrenia. By correlating blood oxygen level-dependent (BOLD) signals in the prefrontal cortex from schizophrenia patients and controls during a working memory task and genotyping data from a genome wide single nucleotide polymorphism (SNP)-array, Potkin et al. (2009) found that the BOLD signal significantly correlated with the presence of two SNPs within the *SLC12A2* gene. Kim et al. (2012) analyzed two independent case control datasets of patients with schizophrenia and healthy controls and found that SNPs in Disrupted-in-Schizophrenia 1 (DISC1) and *SLC12A2* interact epistatically to affect risk for schizophrenia. Finally, one recent study identified a novel heterozygous NKCC1 missense variant in a French-Canadian cohort of schizophrenic patients. Functional studies showed that this variant lead to a gain of function of the transporter (Merner et al., 2016).

Conversely, post-mortem studies in human tissue produced conflicting results. Hyde et al. (2011) found a reduction of KCC2-codying mRNA in the hippocampus, but not dorsolateral prefrontal cortex, of schizophrenic patients. The same study did not find any significant difference in full length NKCC1-codying transcripts. On the other hand Sullivan et al. (2015) reported decreased KCC2 protein expression, by western blot, in the dorsal lateral prefrontal cortex, but not the anterior cingulate cortex, in subjects with schizophrenia. Of note, this group also reported that individuals with schizophrenia off antipsychotic medication at the time of death had decreased KCC2 protein expression compared to both normal controls and subjects with schizophrenia on antipsychotics, suggesting that antipsychotic may affect KCC2 expression levels. Other studies found illness-associated alterations in the expression of NKCC1 transcripts. In particular, Dean et al. (2007) reported an upregulation of NKCC1 mRNA in prefrontal cortex tissue from schizophrenia cases as compared to controls. On the other hand, Morita et al. (2014) analyzed the expression of different NKCC1-codying transcripts produced by alternative splicing and reported reduced expression of the shorter variants of NKCC1 transcripts (NKCC1b and 1-2a) in the dorsolateral prefrontal cortex of subject with schizophrenia compared to a control cohort. Consistently, Zhang et al. (2021) reported reduced NKCC1 mRNA levels in peripheral blood mononuclear cells of patients presenting with the first episode of schizophrenia, and thus not under the effects of antipsychotic medications. Finally, Arion and Lewis (2011) analyzed mRNA expression levels of NKCC1 and KCC2 and their associated regulatory kinases STK39, OXSR1, WNK1, WNK3, and WNK4 in the dorsolateral prefrontal cortex of subjects with schizophrenia compared to non-psychiatric subjects and reported no differences in the expression of either KCC2 or NKCC1 transcripts. However, they found overexpression of OXSR1 and WNK3 transcripts in schizophrenia. These alterations of transcript levels were consistent across subjects with the illness. In addition, they were not altered in monkeys chronically exposed to antipsychotic medications. Together, these findings suggest that the observed difference in OXSR1 and WNK3 levels in schizophrenic individuals were not caused by other factors (medications, substance abuse, etc.). WNK3 is a potent activator of NKCC1, while it can inhibit KCC2 activity (Kahle et al., 2005; de Los Heros et al., 2006). OXSR1 binds to and phosphorylates NKCC1, resulting in an increase of NKCC1 activity (Vitari et al., 2006). Therefore, if increased OXSRI and WNK3 mRNA levels translate into increased protein and thus kinase activity, then this would lead to both enhanced NKCC1 and decreased KCC2 activity, respectively, thus steering neurons toward a predicted higher intracellular Cl^−^ concentration (Arion and Lewis, 2011).

The discrepancy in the findings reported by these studies could be explained in part by differences in the technical approaches used to analyse NKCC1/KCC2 levels and in the medication history of the subjects, by the relative small sample size of each study and heterogeneity of the disease. It is possible that Cl^−^ imbalance may be restricted to specific brain regions, or neuronal circuits, and thus, that only same types of behavioral abnormalities may be affected by targeting Cl^−^ imbalance. More likely, due to the heterogeneity of the diseases, Cl^−^ imbalance mediated by dysregulation of chloride transporters may not be a common pathogenic mechanism in all patients, but it may affect only a subset. If the latter hypothesis is correct, then it is essential to develop and characterize reliable *in vivo* biomarkers for measuring excitation/inhibition imbalance with high spatial and temporal resolution during cognitive tasks. These biomarkers would help stratifying the patients in different populations and identifying those with higher probability of responding to Cl^−^ balance-targeting medications.

## Epilepsy

**Figure 3:**
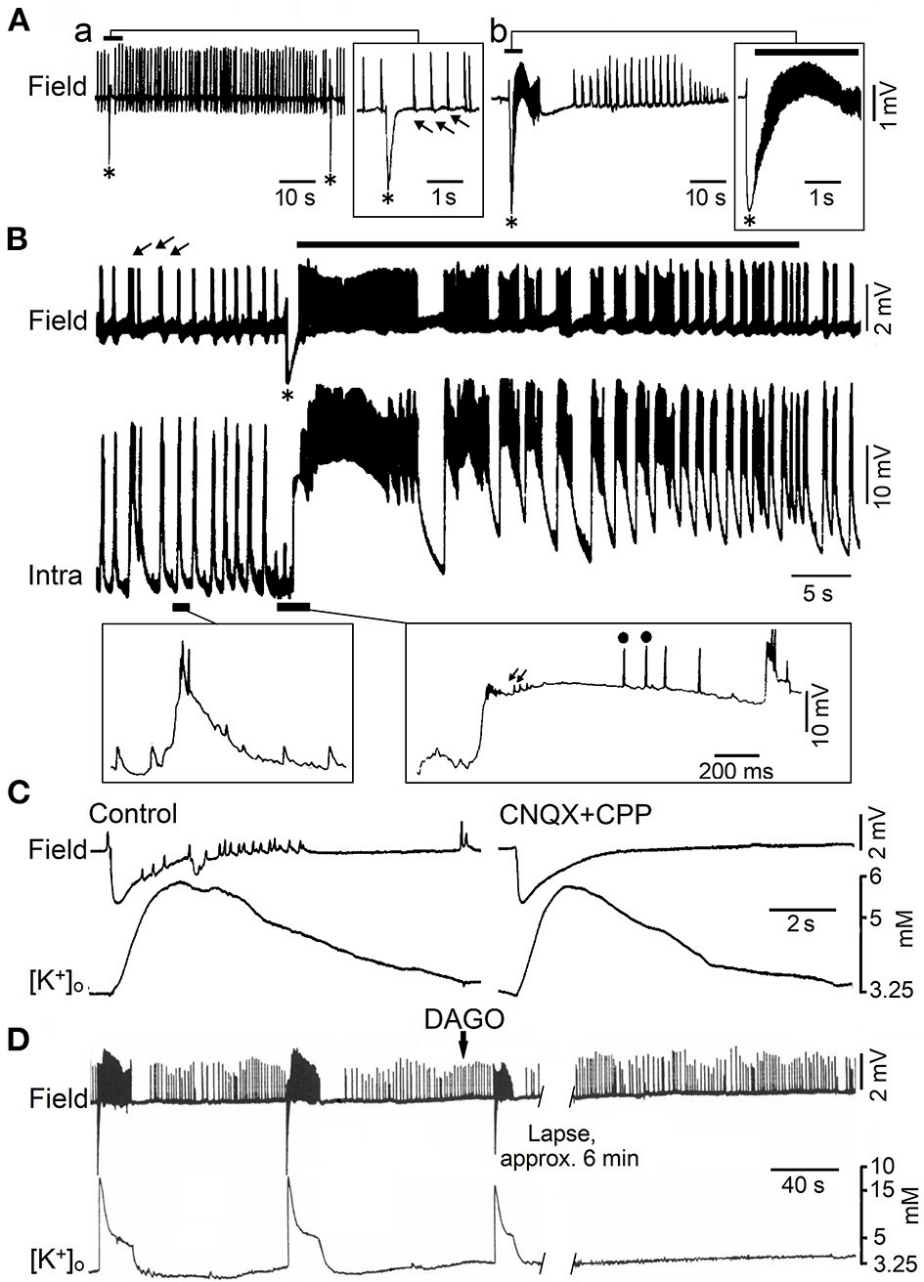
Epileptiform activity induced by 4AP in juvenile rat hippocampal slices maintained *in vitro*. **(A)** Field potential recordings obtained during perfusion with medium containing 4AP from the CA3 subfield of adult (a) and young (14 day-old) brain slices (b). Note that only *fast* (arrows) and *slow* interictal discharges (asterisk) are recorded in adult brain slices while ictal (continuous line) and interictal epileptiform discharges occur in the young hippocampus; note also in (b) that the *slow* (asterisk) interictal discharge leads to the onset of a prolonged ictal event. **(B)** Field and intracellular (Intra) recordings obtained during perfusion with medium containing 4AP from the CA3 area of a 22-day-old rat hippocampal slice; arrows point to the *fast* interictal spikes while asterisk identifies the *slow* interictal that initiates the ictal discharge (thick line). Selected portions of the intracellular recording are illustrated at an expanded time base in the panels below; there, small arrows and dots identify depolarizing potentials and fractionated spikes, respectively. **(C)** Effects induced by application of non-NMDA (CNQX) and NMDA (CPP) receptor antagonists on the 4AP-induced epileptiform activity recorded with field and K^+^ selective microelectrodes from the CA3 subfield of a 19-day-old rat hippocampal slice. Note that this pharmacological procedure blocks the fast interictal events as well as the short-lasting ictal discharge; however the elevation in extracellular [K^+^] that correlates with the negative-going *slow* field potential is not modified. **(D)** Effects of the mu-opioid receptor agonist DAGO on the synchronous activity induced by 4AP in the CA3 subfield of a 15-day-old rat; note that DAGO abolishes the negative-going slow field potential along with the subsequent ictal discharge while fast interictal spikes continue to occur. [**(A)** Is modified from Fueta and Avoli, 1992; **(B)** is modified from Avoli et al. (1993); **(C,D)** are modified from Avoli et al. (1996b)].

Epilepsy is a neurological condition characterized by recurrent spontaneous seizures that can be classified, perhaps in an oversimplified manner, as primary generalized (e.g., those occurring in *absence epilepsy*), and focal (with possible secondary generalization), such as those observed in patients presenting with *mesial temporal lobe epilepsy* (MTLE) or focal cortical dysplasia (Avoli and Gloor, 1987). Here, we will mainly address the role of GABA_A_ receptor signaling in the epileptiform synchronization of limbic networks. Accordingly, focal seizures recorded in MTLE patients arise from limbic structures such as the hippocampus, the amygdala and the rhinal cortices (Gloor, 1997). To note that an epileptic brain with focal abnormalities generates interictal spikes (i.e., short electrographic events <2 s in duration, which are not accompanied by any detectable clinical symptom) (arrows and asterisks in Figures 3Aa,B) and ictal discharges or seizures (i.e., abnormal, hypersynchronous activity lasting up to several tens of seconds, which disrupt normal brain function) (continuous lines in Figures 3Ab, B) (Avoli and Gloor, 1987).

Epilepsy is believed to result from a pathological shift of the E/I balance toward excitation. Accordingly, in the 1950s, clinical studies reported that interfering with GABA synthesis leads to convulsions (Coursin, 1954). In the following decades, *in vitro* experiments have demonstrated that several convulsive drugs are GABA_A_ receptor antagonists (Schwartzkroin and Prince, 1980). In addition, *in vivo* studies have highlighted a decrease of GABAergic inhibition shortly before the onset of electrographic seizures recorded from the hippocampus (Ben-Ari et al., 1979) or neocortex (Kostopoulos et al., 1983). These findings were in line with those indicating that the functional disconnection of interneurons from excitatory inputs impairs inhibition in animal models of focal seizures (Sloviter, 1987), and that alterations in GABA transporter function or in GABA_A_ receptor subunits composition occur in patients affected by focal epileptic disorders and in animal models mimicking them (McDonald et al., 1991; Johnson et al., 1992; Williamson et al., 1995; Brooks-Kayal et al., 1998). Therefore, in the 1990s, weakening of inhibition was considered *a sine qua non conditio* for the occurrence of focal seizures and thus for the establishment of epilepsy.

However, successive studies have challenged this view since epileptiform activity can occur in hippocampal slices maintained *in vitro* during pharmacological manipulations that do not interfere with GABA_A_ receptor mediated inhibition and even enhance it. These experimental procedures include the application of Mg^2+^ free-medium (Mody et al., 1987; Derchansky et al., 2008) or of K^+^ channel blockers such as 4-aminopyridine (4AP) (Voskuyl and Albus, 1985; Rutecki et al., 1987; Perreault and Avoli, 1991, 1992). As illustrated in Figure 3Aa, field potential recordings obtained from the CA3 subfield of adult rat hippocampal slices treated with 4AP, have revealed the occurrence of two distinct types of interictal activity. The first type (arrows in Figure 3Aa), also identified as *fast*, occurs frequently, is driven by CA3 network, and is abolished by non-NMDA glutamatergic receptor antagonists. The second type (asterisks in Figure 3Aa) has a lower rate of occurrence, can initiate in any hippocampal areas, continues to occur when ionotropic glutamatergic excitatory transmission is blocked, and it is abolished by GABA_A_ receptor antagonists (Perreault and Avoli, 1991, 1992) suggesting its GABAergic origin; hence, these 4AP-induced, interictal events have been often referred to as *slow* GABAergic spikes (Perreault and Avoli, 1991, 1992). It has been also found that, in hippocampal slices obtained from young (10–24 day old) rats, 4AP induces ictal discharges (Chesnut and Swann, 1988; Avoli, 1990), which are preceded by a *slow* GABAergic spike (Figure 3Ab, continuous line and asterisk, respectively). Shortly later, intracellular recordings from CA3 pyramidal cells in young hippocampal slices demonstrated that this initial GABAergic spike is characterized by a prolonged depolarization, associated to sparse, fractionated (presumably ectopic) action potentials, leading to the generation of repetitive bursts of action potentials once the ictal activity is established (Avoli et al., 1993) (Figure 3B).

The mechanistic link between such initial synchronous GABAergic event and the onset of electrographic seizure activity in the young rodent CA3 area was supported by findings obtained in successive studies. As illustrated in Figure 3C, the initial GABAergic spikes were mirrored by sizeable elevations in extracellular [K^+^] that were not influenced by application of ionotropic glutamatergic receptor antagonists (Avoli et al., 1996b; see also **Figure 5**). In addition, GABA_A_ receptor antagonists or pharmacological procedures interfering with GABA signaling, such as the application of a μ-type opioid receptor agonist (Capogna et al., 1993), abolished both the initial GABAergic spike and the subsequent ictal activity, that was replaced by on-going, short-lasting interictal spikes (Figure 3D) (Avoli et al., 1996b).

**Figure 4:**
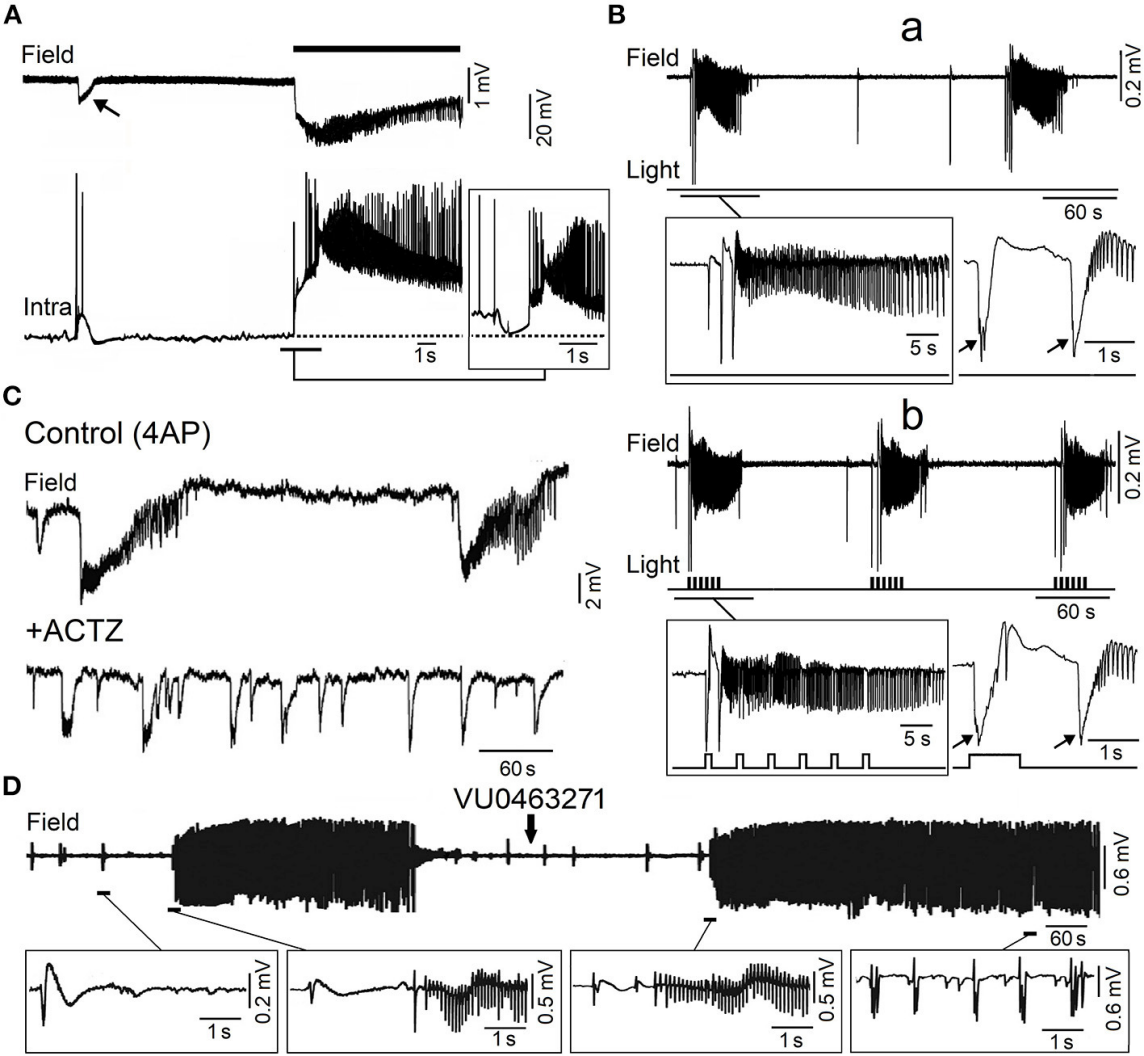
Epileptiform activity induced by 4AP in the adult entorhinal cortex in *in vitro* brain slices. **(A)** Simultaneous field (Field) and intracellular (Intra) recordings obtained from adult rat entorhinal cortex *in vitro* during bath application of 4AP; the recordings shown in the insert were obtained from a different neuron that was depolarized by intracellular current injection to a steady membrane potential at ~62 mV. **(B)** Low voltage fast onset ictal discharges occur spontaneously during bath application of 4AP in the mouse entorhinal cortex (a) but are also triggered by optogenetic stimulation of parvalbumin positive-interneuron (b). The onset of the ictal discharges occurring under each condition is further expanded in the inserts below; note that the ictal discharges are preceded by one or two *slow* interictal spikes (arrows) under both spontaneous and stimulated conditions. **(C)** Field potential recordings obtained from the rat entorhinal cortex under control conditions (4AP) and 20 min after bath application of carbonic anhydrase inhibitor acetazolamide (ACTZ, 10 μM); note that this pharmacological procedure greatly reduces the duration of the ictal discharges. **(D)** Effects of the KCC2 blocker VU0463271 on the 4AP-induced field potential activity recorded from the rat entorhinal cortex; note that addition of VU0463271 induces a pattern of continuous interictal spikes in the field potential recording. Enlarged examples of the field potential recordings are shown below. [**(A)** Is modified from Lopantsev and Avoli (1998); **(B)** is modified from Shiri et al. (2016); **(C)** is modified from Hamidi and Avoli (2015a); **(D)** is modified from Chen et al. (2019)].

Overall, these studies have demonstrated that during application of 4AP, the *slow* GABAergic spikes mostly reflect the synchronous activation of postsynaptic GABA_A_ receptors that leads to increases in extracellular [K^+^] sufficiently large to trigger ictal activity (Morris et al., 1996; Lamsa and Kaila, 1997). Originally, it was proposed that such mechanism only occurs in the juvenile hippocampus because of a decreased homeostasis of extracellular [K^+^] at this early stage of brain maturation (Avoli et al., 1993, 1996b). This hypothesis was, however, not confirmed in successive experiments performed in adult rodent brain slices that could include the entorhinal, perirhinal, piriform, insular cortices, or the amygdala (see for review, Avoli and de Curtis, 2011). In all these cortical structures, 4AP was able to induce *slow* interictal spikes along with ictal discharges that were often preceded by a single (also termed “sentinel”) *slow* GABAergic event (Figure 4A) (see also data obtained from the guinea pig whole brain preparation, Carriero et al., 2010). Seizures with a similar electrographic onset have been recorded with intracranial electrodes in the adult human's brain of patients affected by focal epileptic disorders, including MTLE (Perucca et al., 2014) as well as in *in vivo* animal models (Lévesque et al., 2012).

These ictal discharges have been termed *low-voltage fast onset* seizures since they initiate with a pattern of low voltage oscillatory activity in the *beta*-*gamma* range (see for review Avoli et al., 2016).

As illustrated in Figure 4A, the onset of an ictal discharge recorded *in vitro* in the adult rat entorhinal cortex in the presence of 4AP coincides with principal cell depolarization that, as observed in young rat CA3 pyramidal neurons (*cf*. with insert in Figure 3B), is associated with few “fractionated” (and thus presumptive ectopic) action potentials. Moreover, this initial depolarizing event becomes hyperpolarizing when the neuronal membrane potential is depolarized to values less negative than −60 mV by injecting a steady depolarizing current (Figure 4A; Lopantsev and Avoli, 1998). Indeed, it was known at that time that activation of GABA_A_ receptors can depolarize cortical neurons since these receptors are permeable not only to Cl^−^ but also to ${\text{HCO}}_{3}^{-}$, which has an equilibrium potential more positive than Cl^−^ (Grover et al., 1993; Kaila, 1994). Similar intracellular patterns were also recorded at the onset of 4AP-induced ictal discharges in principal cells of the amygdala in an *in vitro* slice preparation (Benini et al., 2003).

These results, therefore, demonstrate that, paradoxically, the initiation of electrographic seizures occurring *in vitro* coincides with, and thus may be caused by, a robust synchronous inhibitory event. In line with this hypothesis, studies performed by several laboratories have shown that interneurons fire at the onset of these ictal discharges as well as that GABA_A_ receptor antagonists can abolish them (see for review, Avoli and de Curtis, 2011; Avoli et al., 2016). Intense interneuronal firing leading to GABA release and subsequent, excessive activation of GABA_A_ receptors, as identified at the onset of 4AP-induced ictal discharges, coincides also in the adult entorhinal cortex with elevations of extracellular [K^+^] (Avoli et al., 1996a; Librizzi et al., 2017). In turn, these transient increases in extracellular [K^+^] depolarize, and thus recruit neighboring neurons into the synchronous firing that is associated with the ongoing ictal activity (see for review: Avoli and de Curtis, 2011; Avoli et al., 2016). The essential role of interneurons in initiating 4AP-induced ictal discharges was later confirmed by optogenetic studies performed in several laboratories in the entorhinal (Shiri et al., 2015, 2016; Yekhlef et al., 2015) and somatosensory cortex (Chang et al., 2018). As shown in Figure 4B, optogenetic activation of parvalbumin- or somatostatin-positive interneurons initiates ictal discharges with an onset pattern similar to what identified in spontaneously occurring events. Specifically, the onset of both spontaneous and optogenetic-induced ictal discharges is characterized by one-two interictal-like spikes that are followed by fast, *beta*-*gamma* oscillations, i.e., the hallmark of *low-voltage fast* ictal discharges. In line with the participation of inhibitory cells in the initiation of this type of seizure activity, single unit recordings from the seizure onset zone in epileptic patients have shown that GABAergic interneurons increase their firing rate, during the onset of *low-voltage fast* seizures, earlier than excitatory cells (see for review, Weiss et al., 2019). To note, as well, that 4AP-induced electrographic seizures associated with an increase of extracellular [K^+^], presumably due to the high firing rate of interneurons resulting in the activation of postsynaptic GABA_A_ receptors by the massive release of GABA, occur in neocortical slices obtained from epileptic patients with Taylor's type focal cortical dysplasia (D'Antuono et al., 2004; Gigout et al., 2006) but not in brain slices from epileptic patients with no obvious structural abnormalities (Louvel et al., 2001).

**Figure 5:**
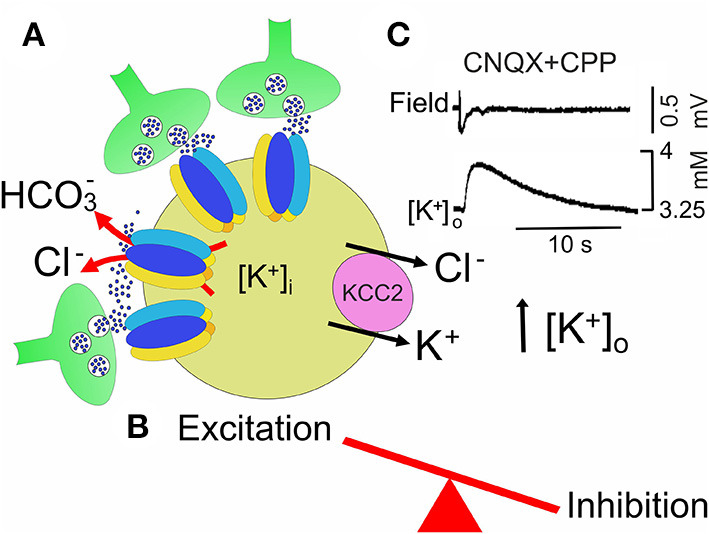
In the 4-AP model of MTLE, neuronal synchronization preceeding ictal dischages, may occur in the absence of glutamatergic transmission. **(A)** GABA, abundantly released from GABAergic interneurons (green) which make synapses with a principal cell (yellow), binds to GABA_A_ receptor-channels, permeable to chloride and ${\text{HCO}}_{3}^{-}$. ${\text{HCO}}_{3}^{-}$ permeability would allow to shift the equilibrium potential for GABA (E_GABA_) toward more positive values respect to ${\text{E}}_{\text{C}{\text{l}}^{-}}$. The high firing rate of GABAergic interneurons, electrically coupled *via* gap junctions, leads to accumulation of K^+^ in the extracellular space (high [K^+^]_o_), which is instrumental in triggering ictal activity. By producing a further shift of E_GABA_ toward more positive potentials, high [K^+^]_o_ contributes to weaken inhibition. In addition, activation of GABA_A_ receptors would cause an inward flux of Cl^−^ that would activate the cation-chloride exporter KCC2. By extruding both Cl^−^ and K^+^, this would further enhance neuronal excitability by further rising [K^+^]_o_ **(B)**. **(C)** Field potential recording of a slow GABAergic event due to the synchronous activation of GABA_A_ receptors (slow GABAergic spike) preceeding elevation of extracellular K^+^ measured with ion-sensitive electrodes in the presence of AMPA and NMDA receptor antagonists, CNQX and CPP, respectively (modified from Avoli et al. (1996a)].

As mentioned above, findings obtained from the *in vitro* 4AP model point at the synchronous postsynaptic activation of GABA_A_ receptors, leading to membrane depolarizations—which are contributed by ${\text{HCO}}_{3}^{-}$ efflux (Figure 5A; Grover et al., 1993; Kaila, 1994)—and to sizable increases in extracellular [K^+^] (Figure 5C; Avoli et al., 1996a,b; Morris et al., 1996; Lamsa and Kaila, 1997), as the fundamental mechanism for triggering *low-voltage fast* ictal activity (Avoli and de Curtis, 2011; Avoli et al., 2016). In line with this hypothesis, application of the carbonic anhydrase inhibitor acetazolamide reduced the duration and the interval of occurrence of ictal discharges induced by 4AP in the piriform and entorhinal cortices (Hamidi and Avoli, 2015a) (Figure 4C). Also to note that Zuckermann and Glaser (1968) discovered over 50 years ago that elevations of [K^+^]_o_ induce neuronal hyperexcitability (Figure 5B) and seizures. Successive studies have demonstrated that the increased extracellular [K^+^] weakens inhibition by causing a positive shift of the reversal of GABA_A_ receptor mediated inhibitory currents (Jensen et al., 1993), and leads to neuronal network resonance, which generates oscillatory patterns in the beta-gamma range (Bartos et al., 2007). It has also been established that activation of GABA_A_ receptors leads to accumulation of intracellular [Cl^−^], which in turn activates KCC2 which extrudes both Cl^−^ and K^+^ from the intraneuronal compartment (Figure 5A; Viitanen et al., 2010). Therefore, the activity of KCC2 may play a role in seizure generation and epileptogenesis (see for review: Di Cristo et al., 2018).

In line with this view, 4AP-induced electrographic ictal discharges recorded from the entorhinal and piriform cortices *in vitro* can be abolished or facilitated by inhibiting or enhancing the activity of KCC2, respectively (Hamidi and Avoli, 2015b; Chen et al., 2019). As illustrated in Figure 4D, the KCC2 antagonist VU0463271 transformed the dynamic pattern of 4AP-induced interictal and ictal activity into a continuous pattern of interictal-like epileptiform events. Similar effects (i.e., the induction of recurrent, robust spiking in both 0-Mg^2+^ and 4AP *in vitro* models) have been reported by Moss' laboratory in the presence of KCC2 antagonists (Sivakumaran et al., 2015; Kelley et al., 2016; Moore et al., 2018). Furthermore, these investigators have found that *in vivo* microinfusion of VU0463271 into the mouse dorsal hippocampus induces recurrent epileptiform discharges thus confirming that KCC2 function is essential for making GABA_A_ receptor mediated inhibition operative (Sivakumaran et al., 2015). More recently, Dzhala and Staley (2021) have identified in an organotypic hippocampal slice model that the KCC2 antagonist VU0463271, by decreasing Cl^−^ extrusion from the intracellular compartment, increases its intracellular elevations during ictal events and discloses a pattern of continuous interictal-like discharges resembling *status epilepticus*. However, in one *in vitro* study performed by Moss' group, KCC2 antagonism could prolong the ictal events induced by 4AP in the entorhinal cortex (Kelley et al., 2016). To note also that Silayeva et al. (2015) have reported that KCC2 activity is essential for controlling *status epilepticus* induced by systemic injections of kainic acid *in vivo*.

*In vitro* electrophysiological recordings from human resected “epileptogenic” brain tissue have demonstrated that the shift of GABA_A_ receptor signaling from hyperpolarizing to depolarizing direction can contribute to network hyperexcitability and neuronal synchronization. Thus, in hippocampal slices from brain tissue removed from patients affected by drug-resistant forms of MTLE, the depolarizing action of GABA in a subset of pyramidal cells (~30%) is present during interictal spikes recorded in the subiculum, the output structure of the hippocampus that projects to the temporal lobe, suggesting a perturbed homeostasis of intracellular [Cl^−^] Cohen et al. (2002). This hypothesis has been confirmed by Huberfeld et al. (2007) who, using combined intracellular recordings and KCC2 immunochemistry, found that subicular cells generating hyperpolarizing responses to GABA were immunopositive for KCC2, whereas cells generating depolarizing responses were immunonegative. Thus, the depolarizing responses to GABA presumably result from excessively high intracellular [Cl^−^] since they were abolished (together with spontaneously occurring field potential events) by bumetanide, a selective blocker of NKCC1; this pharmacological procedure caused a shift of E_GABA_ toward more hyperpolarized values, reinstating GABA_A_-mediated inhibition (Huberfeld et al., 2015). A depolarizing action of GABA, resulting from altered intracellular [Cl^−^] homeostasis, has been also detected in cortical tissue samples obtained from pediatric patients undergoing surgical resection for the treatment of pharmaco-resistant forms of focal epilepsy due to cortical dysplasia (Abdijadid et al., 2015).

An immature depolarizing GABAergic signaling, resulting from a down-regulation of KCC2, has been found also in Scn1b^−/−^ and Scn1a^+/−^ mouse models as well as in brain extracts from patients affected by the Dravet Syndrome, a devastating developmental form of epileptic encephalopathy (Yuan et al., 2019). These data point to KCC2, the major Cl^−^ extruder, as a key determinant for maintaining intracellular [Cl^−^] homeostasis and for regulating neuronal excitability. A rise of intracellular [Cl^−^] following KCC2 dysfunctions impairs GABA_A_-mediated inhibition, with consequent changes in the E/I balance toward excitation, making the brain more prone to seizures. Interestingly, several mutations of the *SLC12A5* gene encoding for KCC2 have been identified in epileptic patients (Duy et al., 2019). Puskarjov et al. (2014b) have reported the R952H mutation of the *SLC12A5* gene in an Australian family with early childhood onset of febrile seizures. In rodent neurons, this mutation led to deficits in neuronal Cl^−^ extrusion associated to impaired formation of cortical dendritic spines. Febrile seizures, which might later lead to idiopathic generalized seizures, have been reported to occur in a French-Canadian cohort carrying both the R952H and the R1049C KCC2 mutations, located in conserved residues of the KCC2 cytoplasmic C-terminus, an important regulatory region of transport function (Kahle et al., 2014).

The role played by the activation of GABA_A_ receptors in promoting epileptiform activity is also supported by the ability of hippocampal networks maintained *in vitro* to generate ictal discharges during pharmacological blockade of both GABA_B_ and ionotropic glutamatergic receptors (Uusisaari et al., 2002). Such unexpected role of GABA_A_ signaling may explain the limited therapeutic efficacy of some antiepileptic drugs that were developed to potentiate GABA_A_ receptor function; these compounds include progabide, γ-vinyl-GABA and tiagabine (Rogawski and Löscher, 2004). Moreover, benzodiazepines—which increase GABA_A_ receptor function in the brain by acting on the allosteric “benzodiazepine site” (Costa et al., 1975; Choi et al., 1977)—can halt seizure activity and *status epilepticus* (Pang and Hirsch, 2005) but do not represent first choice drugs for treating chronic epileptic conditions. It is also worth mentioning that compatible with an excitatory action of GABA, benzodiazepines may paradoxically worsen seizures (Perucca et al., 1998). Bumetanide, by inhibiting NKCC1, may have a beneficial effect, restoring neuronal intracellular [Cl^−^] as demonstrated in the case of a girl affected by epilepsy, cortical dysplasia and ASD (Bruining et al., 2015). Beneficial effects of bumetanide were also detected in a double-blind pilot study on 43 randomized babies affected by neonatal seizures caused by hypoxic-ischemic encephalopathy. This diuretic, added to phenobarbital in dose-escalation design, was able to reduce, in a statistically significant way, seizure burden in the group of subjects treated with phenobarbital and bumetanide (*n* = 27), respect to the control group treated with phenobarbital alone (*n* = 16) (Soul et al., 2021). However, more work is required to establish bumetanide exposure response and safety. Although in the hypoxic-ischemic encephalopathy, the BBB may be compromised (Römermann et al., 2017), usually bumetanide's effectiveness is limited by its poor brain penetration, an effect that can be overcome with new derivatives, currently under development, capable of better permeate the BBB (Savardi et al., 2021).

## Conclusions and Future Perspectives

The discovery of CCC as key regulators of neuronal Cl^−^ concentration has allowed to better understand how GABA, acting on GABA_A_ receptors, influences *via* its depolarizing and excitatory action, several developmental processes whose alterations lead to pathological conditions including ASD, schizophrenia and epilepsy. However, in spite of such progress many questions are still open.

Although bumetanide has shown to have positive effects in a wide range of pathological conditions, its exact mechanisms of action are still poorly understood. In addition, in humans, its beneficial effects are symptomatic and it is unclear whether they are linked to its ability to shift GABA action from the depolarizing to the hyperpolarizing direction by restoring low [Cl^−^]_i_ and a proper E/I balance in neuronal ensembles. The high expression levels of NKCC1 on microglia opens, however new perspectives on the mechanisms of action of this transporter, especially in relation to the role played by activated glial cells in brain inflammation and oxidative stress, which are key features of many neuropsychiatric diseases. Furthermore, to better understand the mechanisms controlling the expression of CCC in the brain and their contribution in shaping, *via* GABAergic signaling, neuronal circuits in both physiological and pathological conditions, it will be crucial to clarify how BDNF/TrkB signaling pathway regulates NKCC1 neuronal expression early in postnatal life (Badurek et al., 2020). An alternative way to re-establish in neurodevelopmental disorders a proper E/I balance by acting on GABA_A_-mediated neurotransmission may be the development of new therapeutic tools selectively targeting the chloride exporters KCC2 which in the CNS are exclusively expressed on neurons (Gagnon et al., 2013; Puskarjov et al., 2014a). By acting either on KCC2 membrane trafficking or on their intrinsic transport kinetics, these compounds will allow attenuating neuronal [Cl^−^]_i_, reinstating an appropriate Cl^−^ homeostasis in selective brain regions. However, new KCC2 activators should not interfere with the well-known KCC2 structural function on dendritic spines formation and dynamics (Blaesse et al., 2009). These new molecules may be particularly useful for treating drug-resistant forms of epilepsy, such as cortical focal dysplasia, occurring in the pediatric age. It is worth mentioning that the use of KCC2 analogs may be limited by their possible proconvulsant effects triggered by accumulation of K^+^ in the extracellular space.

Studies from animal models have allowed identifying early changes in GABAergic signaling as a contributing cause of cognitive deficits observed in Neurodevelopmental Disorders. These are often associated with a loss of particular subtypes of inhibitory interneurons which, by pacing principal cells, give rise to coherent network oscillations, thought to support different behavioral states of the animals and high cognitive tasks. However, with the exception of epilepsy, a direct proof that these processes may occur also in individual affected by neurodevelopmental disorders remains to be demonstrated, making difficult to translate data obtained in preclinical studies from animal models to humans. In the latter case, evidence in favor of an altered GABA_A_-mediated neurotransmission indirectly relies on: (i) genetic studies; (ii) immunohistochemistry from postmortem brain samples showing reduction in GABAergic markers or particular subtypes of GABAergic interneurons; (iii) high incidence of epileptic activity as a comorbidity effect; (iv) alterations in oscillatory activity particularly in the gamma power, detected on the EEG or MEG; and (v) in some cases the paradoxical action of benzodiazepines (for a review see Cellot and Cherubini, 2014b).

An innovative approach consisting in reprogramming human dermal fibroblast obtained through skin biopsy from patients (at progressive stages of the diseases) into induced pluripotent stem cells (iPSCs) and 3D cerebral organoids will provide an innovative platform to uncover disease mechanisms directly in humans. These processes will allow identifying disease mechanisms in a personalized type of manner and to test novel strategies for prevention, diagnosis, patient stratification, therapy and/or rehabilitation.

## Author Contributions

EC, GDC, and MA designed, revised the literature, and wrote the manuscript. EC and MA prepared the Figures. All authors contributed to the article and approved the submitted version.

## Funding

This work was supported by grants from Telethon (GGP 16083) and from Fondo Ordinario Enti (FOE D.M 865/2019) funds in the framework of a collaboration agreement between the Italian National Research Council and EBRI (2019–2021) to EC; from Canadian Institutes for Health Research and Natural Sciences and Engineering Research Council of Canada to GDC; from Canadian Institutes of Health Research (Grants 8109, 74609 and 130328) and the Savoy Foundation to MA.

## Conflict of Interest

The authors declare that the research was conducted in the absence of any commercial or financial relationships that could be construed as a potential conflict of interest.

## Publisher's Note

All claims expressed in this article are solely those of the authors and do not necessarily represent those of their affiliated organizations, or those of the publisher, the editors and the reviewers. Any product that may be evaluated in this article, or claim that may be made by its manufacturer, is not guaranteed or endorsed by the publisher.
